# The Effects of Age on the Human Tear Film Assessed with a Novel Imaging Device

**DOI:** 10.3390/diagnostics15172256

**Published:** 2025-09-06

**Authors:** Alice Verticchio Vercellin, Lauren J. Isserow, Richard B. Rosen, Paul A. Sidoti, Brent A. Siesky, Keren Wood, Nathan Schanzer, Francesco Oddone, Carmela Carnevale, Tak Yee Tania Tai, Masako Chen, Kira Manusis, Katy Tai, David J. Brenner, Norman J. Kleiman, Samuel Potash, George J. Eckert, Gal Antman

**Affiliations:** 1 Ophthalmology, Icahn School of Medicine at Mount Sinai, New York, NY 10029, USA; li81@rwjms.rutgers.edu (L.J.I.); rrosen@nyee.edu (R.B.R.); psidoti@nyee.edu (P.A.S.); brent.siesky@mssm.edu (B.A.S.); keren.woodshalem@mssm.edu (K.W.); nathan.schanzer@mssm.edu (N.S.); ttai@nyee.edu (T.Y.T.T.); masako.chen2@mountsinai.org (M.C.); kmanusis@nyee.edu (K.M.); samuel.potash@mssm.edu (S.P.); antmangal@gmail.com (G.A.); 2 Ophthalmology, Rutgers Robert Wood Johnson Medical School, New Brunswick, NJ 08901, USA; 3 Ophthalmology, New York Eye and Ear Infirmary of Mount Sinai, New York, NY 10003, USA; ktai@nyee.edu; 4 New York Medical College, Valhalla, NY 10595, USA; 5 IRCCS Fondazione G B Bietti per lo Studio e la Ricerca in Oftalmologia ONLUS, 00184 Roma, Italy; francesco.oddone@fondazionebietti.it (F.O.); carmela.carnevale@fondazionebietti.it (C.C.); 6 Center for Radiological Research, Columbia University Irving Medical Center, New York, NY 10032, USA; djb3@cumc.columbia.edu; 7 Environmental Health Sciences, Mailman School of Public Health, Columbia University, New York, NY 10032, USA; njk3@cumc.columbia.edu; 8 Department of Biostatistics and Health Data Science, Indiana University School of Medicine and Richard M. Fairbanks School of Public Health, Indianapolis, IN 46202, USA; geckert@iu.edu; 9 Faculty of Medicine, Tel Aviv University, Tel Aviv 69978, Israel; 10 Ophthalmology, Rabin Medical Center, Petah Tikva 4941492, Israel

**Keywords:** tear film, age, tear film imager, muco-aqueous layer thickness (MALT), lipid-layer thickness (LLT)

## Abstract

**Purpose:** We aimed to analyze the effects of age on human tear film (TF) using a novel nanometer resolution TF imaging device (Tear Film Imager, TFI, AdOM, Israel). **Methods:** 44 healthy adult subjects (≥18 years of age) without ocular or systemic diseases or prior eye treatments with ages spanning seven decades were enrolled in this prospective cross-sectional study. Subjects underwent a comprehensive ophthalmic examination and completed the Ocular Surface Disease Index questionnaire (OSDI). All study participants underwent TF imaging using the TFI, including assessment of muco-aqueous layer thickness (MALT), lipid-layer thickness (LLT), inter-blink interval, and lipid map uniformity. Associations between TFI parameters and age were tested using linear regression (accounting for multiple eyes). **Results:** A total of 80 eyes (44 subjects) were imaged: 19 eyes from 10 subjects in the 3rd decade of life (aged 20–29); 10 eyes from 5 subjects in the 4th decade of life (aged 30–39); 5 eyes from 3 subjects in the 5th decade of life (40–49); 12 eyes from 7 subjects in the 6th decade of life (50–59), 19 eyes from 11 subjects in the 7th decade of life (60–69); 11 eyes from 6 subjects in the 8th decade of life (70–79); and 4 eyes from 2 subjects in the 9th decade of life (80–89). With increasing age, MALT significantly decreased (*p* = 0.024), and LLT significantly increased (*p* = 0.001). No statistically significant linear age effects were found for the other TFI parameters (*p* > 0.05) or the OSDI scores of study participants of different ages (*p* = 0.786). **Conclusions:** Quantitative TF biomarkers varied significantly with advancing age in healthy individuals, highlighting the importance of accounting for age in TF assessments.

## 1. Introduction

The tear film (TF) is a multi-layered liquid covering the anterior corneal surface, containing muco-aqueous and lipid sublayers [1,2]. TF is essential for maintaining ocular health and plays a critical role in protecting the eye from environmental insults, lubricating the eye’s anterior surface, and providing antimicrobial protection [1,3,4]. Proper function of the tear film layer is essential for vision and refraction [5,6]. Stability and maintenance of its superficial lipid layer, produced by the Meibomian glands [2], and its deep muco-aqueous layer, produced by the lacrimal glands, are important in preventing ocular surface diseases such as dry eye and keratoconjunctivitis sicca [2,7,8]. Moreover, proper closure of the lids and lid margins, as well as blink mechanisms, are also essential for maintaining TF stability and ocular health [7].

Patient complaints related to TF are evaluated with ocular questionnaires, while fluorescein tear break-up time and the Schirmer test are used in clinical practice to assess TF stability and production [1,9]. While these methods are widely used clinically, they fail to demonstrate objective quantitative information about TF sublayers [9]. Recently, TF imagers (such as the Oculus Keratograph 5M, the LipiView interferometer, and the Ocular Surface Analyzer) have been developed to quantitatively assess TF [10,11,12]. The Tear Film Imager (TFI) (AdOM, Israel), however, is the only device capable of measuring both the static and dynamic qualities of TF sublayers comprehensively with nanometer resolution [10].

Age affects the physiology of many bodily functions, and several studies have investigated the effects of age on certain aspects of the human TF [13]. Reductions in tear volume, increased TF osmolarity, and reduced TF stability have been reported to be associated with older age [13,14]. Specifically, age-related dysfunction of the lacrimal glands may contribute to reductions in tear volume [15]. Other studies in the same population suggest that Meibomian gland dysfunction, which is more common in older adults, is associated with dry eye disease [13,16]. The literature is not all in agreement; as other studies demonstrate, no differences in TF quality or pathologic characteristics, such as altered stability or osmolarity, are a function of age [17]. For example, in a study of healthy contact lens wearers, no age-related difference in TF break-up time was reported [18]. These previous studies, however, utilized qualitative methods for TF evaluation, including fluorescein staining, interference patterns, and other non-quantitative methods [18,19]. The lack of objective quantitative data highlights the need for objective quantitative data on the effects of age on TF characteristics. Moreover, previous studies did not examine individual sublayers of the human TF quantitatively with respect to age. There is a knowledge gap in our current understanding of the relationship between physiological aging and TF changes in healthy subjects [13,20].

To the best of our knowledge, this manuscript is the first to quantitatively investigate the effects of age on TF in healthy adults without any history of ocular or systemic disease or ocular therapies. The objective of this study is, therefore, to quantitatively measure human TF sublayers in healthy subjects whose ages range across seven decades using a novel TFI device.

## 2. Materials and Methods

In total, 80 eyes from 44 subjects were evaluated in a prospective cross-sectional pilot study conducted between February 2023 and July 2025 at The New York Eye and Ear Infirmary of Mount Sinai, New York, NY. Written informed consent was obtained from participants prior to participation in this study. This study was conducted in accordance with the Declaration of Helsinki and was approved by the Institutional Review Board at the Icahn School of Medicine at Mount Sinai, New York, NY (STUDY-22-01415).

Inclusion criteria included patients aged 18 years or older with no history of ocular disease or ophthalmic surgical or medical treatments. Study participants were excluded if they presented with ocular symptoms, were treated with any ocular medications (including artificial tears), underwent any previous eye surgery, presented any systemic disease that could affect TF (i.e., Sjogren disease), or were contact lens wearers.

**Figure 1:**
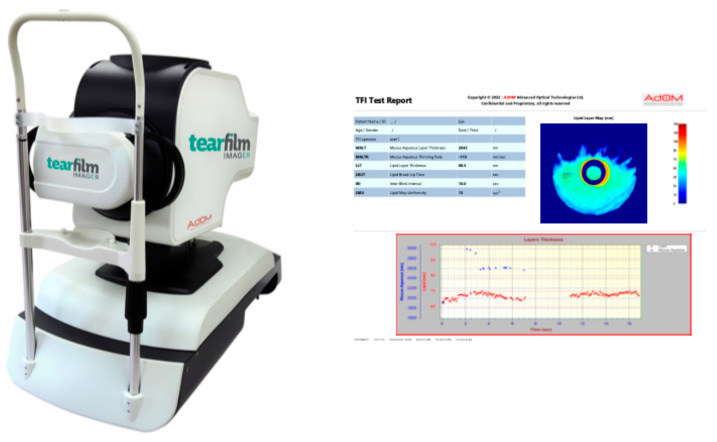
Tear Film Imager (TFI) device (**left**) and TFI test report (**right**).

All study participants’ ocular health was evaluated comprehensively by a cornea specialist. All study subjects completed the Ocular Surface Disease Index (OSDI) questionnaire, a 12-question validated questionnaire used to assess dry eye symptoms [21]. Participants then underwent a non-contact assessment of their TF using the novel, non-invasive TFI (software version 3.11, AdOM, Israel), which uses spectral interference to image TF sublayers with nanometer resolution [22]. Briefly, the TFI provides a 40 s non-invasive quantitative assessment of the dynamic and static TF sublayers, including the muco-aqueous layer thickness (MALT), lipid-layer thickness (LLT), inter-blink interval (IBI), and lipid map uniformity (LMU) (Figure 1). Room temperature and humidity were recorded in testing rooms using a combined thermometer and hygrometer (Govee Hygrometer Thermometer, model H5075, China). TFI images were captured by one of three trained imagers. Only high-quality images, as demonstrated by image quality parameters (test duration, signal score, position score, and data continuity score), were included for analysis.

Statistical calculations were performed using SAS version 9.4 software (SAS Institute, Inc., Cary, NC, USA). Summary statistics were calculated by age group. A generalized additive model was used to visualize the association of age with TFI parameters and examine the relationships for nonlinear effects. Linear regressions, calculated using linear mixed effects models to account for the inclusion of both eyes for some subjects, were used to test for linear associations between age and TFI parameters. A two-sided 5% significance level was used for all tests.

## 3. Results

### 3.1. Participant Demographics

Eighty eyes from 44 patients were included in this study. Participants’ ages spanned 7 decades from the 3rd to the 9th decade. In total, 10 participants (19 eyes) were in the 3rd decade of life (age 20–29), 5 participants (10 eyes) were in the 4th decade of life (ages 30–39), 3 participants (5 eyes) were in the 5th decade of life (ages 40–49), 7 participants (12 eyes) were in the 6th decade of life (ages 50–59), 11 participants (19 eyes) were in the 7th decade of life (ages 60–69), 6 participants (11 eyes) were in the 8th decade of life (ages 70–79), and 2 participants (4 eyes) were in the 9th decade of life (ages 80–89. Eighteen (40.9%) patients were female, and twenty-six (59.1%) patients were male. In total, 5 (11.4%) patients identified as Asian, 6 patients (13.6%) identified as Black, 1 (2.3%) patient identified as Middle Eastern, one (2.3%) patient identified as multiracial (White, Asian), 18 (40.9%) patients identified as white, 5 (11.4%) patients identified as Hispanic, and 8 (18.2%) patients did not specify their race. There were no statistically significant differences between TFI biomarkers in male and female genders (*p* > 0.5).

### 3.2. OSDI Scores and TFI Biomarkers

Ocular Surface Disease Index scores and TFI biomarker results (mean and SD) are shown in Table 1.

**Figure 2:**
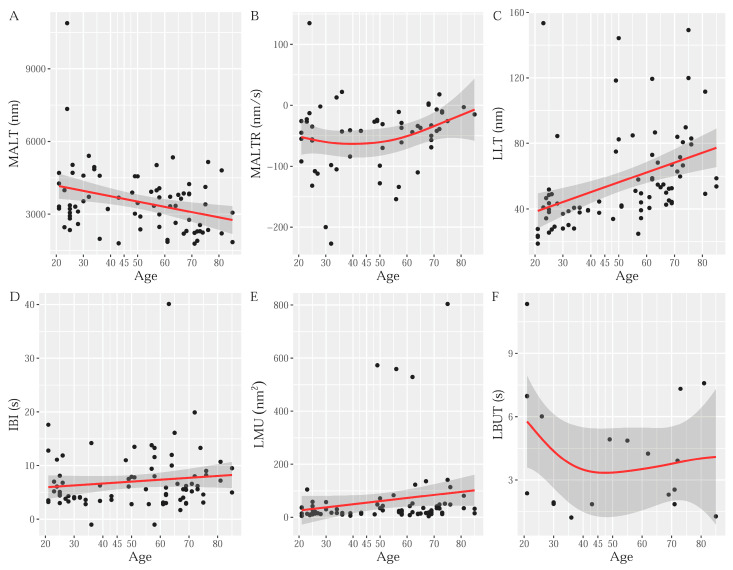
Scatterplots with generalized additive model-fitted lines and confidence intervals for Tear Film Imager (TFI) parameters versus age. Red lines indicate fitted regression models, and gray shaded areas indicate 95% confidence intervals. (**A**) Muco-aqueous layer thickness (nm) vs. age (years); (**B**) muco-aqueous layer thinning rate (nm/s) vs. age (years); (**C**) lipid layer thickness (nm) vs. age (years); (**D**) inter-blink interval (s) vs. age (years); (**E**) lipid map uniformity (nm^2^) vs. age (years); and (**F**) lipid break-up time (s) vs. age (years).

With increasing age, there is a statistically significant decrease in MALT (*p* = 0.024) and a significant increase in LLT (*p* = 0.001, Table 1; Figure 2A,C). No significant linear age effects were observed for OSDI scores, MALTR, IBI, LBUT, LMU, or TFI parameters regarding test quality (test duration, signal score, position score, and data continuity score), as shown in Table 1 and Figure 2B,D–F.

## 4. Discussion

TF is vitally important for maintaining ocular health, defense against environmental insults, protection against infection, and visual function [1,2,5]. Evidence for any effect of age on human TF is mixed and lacks objective quantitative data on TF sublayers. In contrast, this manuscript reports age-related changes in some aspects of TF using a novel TFI instrument [22] that quantitatively measures TF sublayers at nanometer resolution in healthy participants whose ages span 7 decades.

Our findings demonstrated progressive thinning of the MALT with age (*p* = 0.024), as shown in Table 1 and Figure 2A. Previous studies have demonstrated that increasing age is associated with a reduction in Schirmer test tear production and tear fluid volume [13,17]. These observations are likely due to a reduction in lacrimal gland function and subsequent TF production that physiologically occurs with age [17]. Deficiencies in tear production lead to reductions in MALT and TF stability [17]. In contrast to the findings of this study, other reports did not find significant age-related differences in TF stability and evaporation, most likely due to the non-quantitative methodologies used and small sample sizes [17]. No previous studies quantitatively assessed changes in MALT as a function of age. Future TFI studies with larger numbers of subjects would be useful to more fully describe the relationship between age and MALT.

Our results show progressive thickening of the LLT associated with age (*p* = 0.001), as shown in Table 1 and Figure 2C. This finding is likely due to Meibomian gland dysfunction (MGD), which is an ocular pathology affecting up to 56% of adults between the ages of 40 and 80 years [23]. Meibomian gland dysfunction affects lipid production, leading to increased TF osmolarity and thicker LLT [17]. As all our study participants were free from any ocular disease (and denied any ocular symptoms), these findings seem to suggest that MGD may be asymptomatic and affect TF without causing visual discomfort. The TFI may, therefore, represent an innovative methodology to detect asymptomatic MGD affecting TF layers, but not causing dry eye symptoms. In this context, previous studies on patients with MGD and dry eye disease demonstrated a positive association between LLT and age, as measured via the Lipiview Interferometer [24,25]. While these studies demonstrate a similar trend to the current study, none included healthy participants or used the TFI. Additional studies focusing on healthy participants producing quantitative outcomes are needed to further understand connections between age and LLT.

Our study did not find significant differences in the TFI biomarkers (MALTR, IBI, LMU, and LBUT) and quality parameters (test duration, signal score, position score, and data continuity score) among different age groups (*p* > 0.05), as shown in Table 1 and Figure 2B,D–F. The OSDI score was normal across all decades, and we did not find any significant age-related differences (*p* = 0.786, Table 1). While older patients are at higher risk for dry eye disease (DED) and related symptoms, all participants in this study were free of clinically evident ocular disease, including DED [17]. In a previous study by Di Zazzo et al. on healthy volunteers who self-reported ocular health, the authors found that increased OSDI was associated with increasing age [26]. In contrast to that study, which did not include any ocular exam prior to enrollment, it is important to highlight that prior to inclusion in our study, patients were required to undergo a comprehensive ocular exam to be eligible for participation. Patients without known DED, but with clinical signs of DED, may have been inadvertently included in the Di Zazzo study but would have been excluded in the present study. Importantly, in our study, the TFI was able to detect differences in the MALT and LLT sublayers in participants of different ages without any ocular symptoms. This suggests that TFI imaging may provide a quantitative method to non-invasively assess TF in asymptomatic patients. These findings highlight how the TFI can be used to provide quantitative data on TF health and relevant clinical parameters in healthy participants of all ages. Tear film imaging using the TFI may provide evidence of subclinical, asymptomatic DED, identifying patients at risk for this condition. Future longitudinal studies will help to better define its potential.

This study has several limitations, including a relatively small sample size of 44 individuals and unequal numbers of inclusions across age groups. Despite this limitation, our findings demonstrate significant age-related differences in MALT and LLT. Our study also includes patients with dry eyes, as the main objective was to study the effect of age on TF under normal physiological conditions. Our findings are designed to build a foundation for future studies assessing the differences in TF sublayers between healthy and DED patients. While this study includes patients with a variety of racial and ethnic backgrounds, most participants in this study identified as Caucasian. Race is a factor in DED symptomology and physical presentation, likely due to many factors, including differences in eye anatomy and societal and health disparities [27,28]. We did not find differences in TFI parameters as a function of gender. Other studies report mixed evidence for the effect of gender on the sublayers of TF [17,29]. Larger numbers of subjects with racial and ethnic diversity may help resolve any uncertainties about the relationship between TF, physiological aging, gender, and race.

## 5. Conclusions

This pilot study is the first to show that age significantly affects TF sublayer biomarkers in individuals without any ocular disease. With increasing age, there is a decrease in MALT and an increase in LLT. In contrast, OSDI scores were not associated with subject age. These findings suggest that reductions in aqueous layer production due to aging lacrimal glands and alterations in lipid production due to aging Meibomian glands may contribute to asymptomatic changes in TF in healthy adult patients without known ocular disease. Age-related changes in TF sublayers are detectable with a non-invasive nanometer resolution TFI and may be important for diagnosing risks for DED.

## Figures and Tables

**Table 1:** OSDI scores and Tear Film Imager (TFI) biomarkers (mean and standard deviation) in the study population for each decade of age. Number of participants, number of eyes, and mean (standard deviation).

Parameter	Age	Number of Participants	Number of Eyes	Mean	Standard Deviation	*p*-Value
OSDI Score	20–29	10	10	6.9	9.8	0.786
	30–44	6	6	6.5	7.7	
	45–59	9	9	11.3	12.9	
	60–69	10	10	10.5	18.1	
	70+	8	8	3.2	7.2	
MALT (nm)	20–29	10	19	4009	2047	0.024 **
	30–44	6	12	3797	1146	
	45–59	9	15	3627	779	
	60–69	10	17	3338	986	
	70+	8	15	2819	1078	
MALTR (nm/s)	20–29	10	16	−46.6	61.6	0.164
	30–44	6	10	−82.9	83.2	
	45–59	8	13	−64.1	49.1	
	60–69	6	10	−42.9	32.9	
	70+	6	9	−15.1	18.6	
LLT (nm)	20–29	10	19	44.6	30.4	0.001 **
	30–44	6	12	36.8	5.3	
	45–59	9	15	61.1	33.7	
	60–69	11	19	59.0	19.0	
	70+	8	15	81.6	27.7	
IBI (s)	20–29	10	19	6.56	4.03	0.275
	30–44	6	12	4.44	3.49	
	45–59	9	15	8.01	4.31	
	60–69	11	19	9.27	10.99	
	70+	8	15	8.23	4.22	
LMU (nm^2^)	20–29	10	18	26.4	24.2	0.161
	30–44	6	12	20.3	13.8	
	45–59	9	15	108.5	190.5	
	60–69	11	19	59.0	119.5	
	70+	8	15	99.6	199.2	
LBUT (s)	20–29	3	5	6.71	3.24	0.530
	30–44	3	4	1.66	0.38	
	45–59	2	2	4.90	0.04	
	60–69	2	2	3.28	1.38	
	70+	5	6	4.45	2.90	
Test Duration (s)	20–29	10	19	18.2	9.3	0.426
	30–44	6	12	21.0	8.2	
	45–59	9	15	19.3	7.2	
	60–69	11	19	14.4	9.2	
	70+	8	15	19.0	9.2	
Signal Score (%)	20–29	10	19	0.037	0.014	0.122
	30–44	6	12	0.038	0.011	
	45–59	9	15	0.040	0.016	
	60–69	11	19	0.032	0.016	
	70+	8	15	0.029	0.008	
Position Score	20–29	10	19	9.9	5.6	0.953
	30–44	6	12	11.2	5.6	
	45–59	9	15	11.0	7.5	
	60–69	11	19	11.4	5.8	
	70+	8	15	9.7	5.7	
Data Continuity Score (s)	20–29	10	19	0.98	1.64	0.057
	30–44	6	12	0.85	1.20	
	45–59	9	15	1.04	1.44	
	60–69	11	19	0.38	0.56	
	70+	8	15	0.31	0.63	

OSDI: Ocular Surface Disease Index, MALT: muco-aqueous layer thickness, MALTR: muco-aqueous layer thinning rate, LLT: lipid layer thickness, IBI: inter-blink interval, LMU: lipid map uniformity, LBUT: lipid break-up time. The test duration, signal score, position score, and data continuity score are measures of image quality. ** indicates statistical significance, *p* < 0.05.

## Data Availability

The data presented in the study are included in the article; further inquiries can be directed to the corresponding author.
